# Effect of low versus high balance training complexity on balance performance in male adolescents

**DOI:** 10.1186/s13104-024-06811-x

**Published:** 2024-05-28

**Authors:** Thomas Muehlbauer, Lucas Eckardt, Lukas Höptner, Mathew W. Hill

**Affiliations:** 1 Division of Movement and Training Sciences, Biomechanics of Sport, University of Duisburg- Essen, Essen, Germany; 2 Centre for Physical Activity, Sport and Exercise Sciences, Coventry University, Coventry, UK

**Keywords:** Postural control, Adolescence, Intervention, Training modality, Dose-response relationship

## Abstract

**Objective:**

The current study aimed to determine the effects of low (i.e., balance task only) versus high (i.e., balance task combined with an additional motor task like dribbling a basketball) balance training complexity (6 weeks of training consisting of 2 × 30 min balance exercises per week) on measures of static and dynamic balance in 44 healthy male adolescents (mean age: 13.3 ± 1.6 years).

**Results:**

Irrespective of balance training complexity, significant medium- to large-sized pretest to posttest improvements were detected for static (i.e., One-Legged Stance test, stance time [s], 0.001 < *p* ≤ 0.008) and dynamic (i.e., 3-m Beam Walking Backward test, steps [*n*], 0.001 < *p* ≤ 0.002; Y-Balance-Test-Lower-Quarter, reach distance [cm], 0.001 < *p* ≤ 0.003) balance performance. Further, in all but one comparison (i.e., stance time with eyes opened on foam ground) no group × test interactions were found. These results imply that balance training is effective to improve static and dynamic measures of balance in healthy male adolescents, but the effectiveness seems unaffected by the applied level of balance training complexity.

## Introduction

There is evidence that balance training (BT) is an effective method to improve static and dynamic balance in adolescents [1–3]. Schedler et al. [1] detected improved 10-m gait velocity and reach distance for the Y-Balance-Test-Lower-Quarter (YBT-LQ) test following five weeks of BT in adolescents. Pau et al. [2] found significantly reduced postural sway area for the bipedal stance test in adolescent volleyball players after six weeks of BT. Decreasing reflex activities [4] as well as structural and functional brain changes [5, 6] are stated as the underlying mechanisms of these balance improvements.

Despite the positive findings, the question arises how to design BT effectively but also efficiently in youth. Due to restricted time resources in schools (P.E. lessons) and sports clubs, increasing the duration, frequency or volume of BT has low practical relevance. In contrast, an increase in BT difficulty (e.g., modified sensory demands), intensity (e.g., modified motor demands) or complexity (e.g., simultaneous execution of several motor tasks) does not require additional time resources [7]. Concerning BT difficulty, Schedler et al. [7] applied seven weeks of BT in male adolescents and observed greater improvements in the Functional-Reach test (FRT) distance, the YBT-LQ reach distance, and the One-Legged Stance test (OLS) time for the those practising with a high (e.g., bipedal, tandem, and unipedal stance exercises with eyes closed) compared to those with a low (e.g., bipedal, tandem, and unipedal stance with eyes opened) task difficulty level. Regarding BT intensity, Blasco et al. [8] conducted BT (e.g., cross-over steps on stable versus unstable ground) over three weeks in young adults and observed similar improvement (i.e., YBT-LQ and FRT reach distance, OLS time), irrespective whether the groups trained on stable (means low intensity level) or unstable (means high intensity level) ground. Despite these gains in knowledge, it is unknown if the reported effects of BT intensity and difficulty on balance performance generalise to BT complexity (i.e., dual tasking). In fact, there is evidence [9, 10] showing that dual- compared to single-task practice is suitable to free up central resources that can be used for a more effective processing of postural control resulting in larger improvements.

We aimed to compare the effect of a six-weeks BT program with low (i.e., single-motor-tasking) versus high (i.e., dual-motor-tasking) level of complexity on static and dynamic balance in healthy male adolescents. Given the previous findings, we hypothesised that both training regimens would improve balance. Further, we expected that a high versus low complex BT program would lead to greater improvements. The investigation of male adolescents is particularly relevant, as they show poorer balance performance than aged-matched females [11, 12]. This finding is explained by the delayed maturation of the postural control system [13, 14]. This can result in an increased risk of falls and associated injuries [15], which in turn can be very costly to treat medically [16].

## Main text

### Methods

#### Participants

Previous research [17] has reported medium- to large-sized effect sizes that were used for sample size estimation. Precisely, a G*power analysis (effect size *f* = 0.25, α error probability = 0.05, *1-β* error probability = 0.80, correlations among repeated measures *r* = 0.40, 2 groups, 2 assessments, drop-out rate of 10% due to reasons not attributable to treatment) revealed that a total sample size of *N* = 40 participants (i.e., *n* = 20 per group) would be sufficient to detect significant treatment effects [18]. Thus, 44 healthy, male, physically active adolescents participated in this study and were randomly assigned to the BT-low-complex group (i.e., single-motor-tasking) or the BT-high-complex group (i.e., dual-motor-tasking) (Table 1). The participants were recruited via an information event from public secondary schools in the Ruhr area of North Rhine-Westphalia, Germany. All individuals participated were free of any neurological or musculoskeletal impairment.

**Table 1:** Means ± standard deviations for the demographic characteristics of the sample by group

Characteristic	BT-low-complex (*n* = 22)	BT-high-complex (*n* = 22)	*p*-value
Chronological age [years]	13.2 ± 1.6	13.5 ± 1.6	0.512
Biological age [years from PHV]	0.09 ± 1.44	0.28 ± 1.37	0.660
Body height [cm]	169.0 ± 11.9	169.1 ± 11.9	0.980
Body mass [kg]	56.5 ± 11.4	59.6 ± 14.8	0.436
Body mass index [kg/m²]	19.6 ± 2.2	20.6 ± 3.2	0.238
Leg length [cm]	90.6 ± 6.1	91.2 ± 6.2	0.734

*BT* Balance training; *PHV* peak height velocity

#### Study design and experimental protocol

This randomized-parallel trial consisted of a pretest and a posttest that were separated by a six-weeks treatment period. Upon entering the laboratory, all participants received standardised verbal instructions and visual demonstrations regarding the balance assessment.

#### Assessment of static balance performance

The same skilled assessors conducted the assessment of balance before and after the training period. Static balance was assessed using the timed OLS test. Participants were asked to stand without shoes on their non-dominant leg (determined by self-report) for as long as possible but for a maximum of 60 s. The OLS was conducted in four different conditions: (1) eyes opened on firm ground (EO-FI), (2) eyes closed on firm ground (EC-FI = supressed vision/proprioception dominant), (3) eyes opened on foam (i.e., AIREX balance pad) ground (EO-FO = modified proprioception/vision dominant), (4) eyes closed on foam ground (EC-FO = supressed vision and modified proprioception/vestibular dominant). After a practice trial, one data-collection trial was executed, and the maximal stance time (s) during each condition was used for further analysis. The timed OLS test is valid (concurrent and discriminative) and reliable (moderate to excellent) in youth [19, 20].

#### Assessment of dynamic balance performance

Dynamic balance was determined using the 3-m Beam Walking Backward test [21] that consists of wooden beams (length: 3 m; height: 5 cm) that differed in width (i.e., 6.0, 4.5, 3.0 cm). The participants wore sports shoes and were asked to walk backward at a self-selected speed from the beginning to the end of each beam but for a minimum of eight steps. One practice trial followed by two data-collection trials per beam width were performed. The step number for the data-collection trials was added up resulting in a maximum of 16 steps per beam width and 48 steps in total. Dynamic balance was further assessed using the YBT-LQ. While maintaining a one-legged stance with the non-dominant limb on the central footplate, participants were asked to reach with their dominant leg as far as possible in the anterior (AT), posteromedial (PM), and posterolateral (PL) directions. For each direction, one practice trial followed by three data-collection trials were performed and the greatest reach distance (cm) per direction was used for subsequent analysis. Specifically, these values were normalized (% leg length [LL]) by dividing maximal reach distance by LL and then multiplying by 100. Further, the composite score (CS in % LL) was calculated as the sum of the maximal reach distance per direction divided by three times LL and then multiplied by 100. The LL of each participant was determined from the anterior superior iliac spine to the most distal aspect of the medial malleolus [22]. The YBT-LQ test is valid (concurrent, discriminative, and predictive) and reliable (moderate to excellent) in youth [23–25].

#### Balance training

Both groups received BT for six weeks (i.e., 2 × 30 min/week) that was supervised by graduated students. Each session included three balance exercises (i.e., unipedal stance, 3-m balancing walk, and unipedal jump-landings) and each of them was performed four times (Table 2). The BT-low-complex group performed the exercises as single-task (i.e., balance task only) and the BT-high-complex group as dual-task (i.e., balance task combined with dribbling a basketball/handball or throwing/catching/heading a ball) [26, 27]. Training progression was achieved by minimising the base of support diameter of a balance board (Wobblesmart©, Artzt GmbH, Dornburg, Germany) from level 1 (week 1) to level 6 (week 6), reducing the balance beam width from 6.0 cm (week 1–2) over 4.5 cm (week 3–4) to 3.0 cm (week 5–6), alternating the walking direction, and changing the landing surface from firm to foam.

**Table 2:** Description of the balance training program by complexity level

Exercise	Week 1	Week 2	Week 3	Week 4	Week 5	Week 6
Unipedal stance^1^	4 × 60 s per leg; level 1	4 × 60 s per leg; level 2	4 × 60 s per leg; level 3	4 × 60 s per leg; level 4	4 × 60 s per leg; level 5	4 × 60 s per leg; level 6
3-m balancing walk^1^	4 × 60 s fw; 6 cm width	4 × 60 s bw; 6 cm width	4 × 60 s fw; 4.5 cm width	4 × 60 s bw; 4.5 cm width	4 × 60 s fw; 3 cm width	4 × 60 s bw; 3 cm width
Unipedal jump-landings	4 × 60 s per leg; firm, throw^2^	4 × 60 s per leg; foam, throw^2^	4 × 60 s per leg; firm, catch^3^	4 × 60 s per leg; foam, catch^3^	4 × 60 s per leg; firm, header^4^	4 × 60 s per leg; foam, header^4^

Level 1–6 refers to a balance board (Wobblesmart©, Artzt GmbH, Dornburg, Germany) with a mechanically adjustable pivot to increase task difficulty from level 1 (low) to 6 (high) by reducing the base of support diameter. ^1^Dribbling a basketball/handball; ^2^Throwing a ball; ^3^Catching a ball; ^4^Heading a ball. *bw* backward; *fw* forward

#### Statistical analyses

Data were reported as group means ± standard deviations. After normal distribution was confirmed (i.e., Shapiro-Wilk tests), a two-way (group: BT-low-complex, BT-high-complex × test: pretest, posttest) repeated measures analysis of variance (ANOVA) was conducted to detect training-related group differences. Where significant interactions were detected, post-hoc analyses using Bonferroni-adjusted α determined the location of any differences. Additionally, effect size (*η*_p_^2^) was calculated and reported as small (0.02 ≤ *η*_p_^2^ ≤ 0.12), medium (0.13 ≤ *η*_p_^2^ ≤ 0.25), or large (*η*_p_^2^ ≥ 0.26) [28]. For the post-hoc analyses, the effect size Cohen’s *d* was determined and interpreted as trivial (0 ≤ *d* ≤ 0.19), small (0.20 ≤ *d* ≤ 0.49), moderate (0.50 ≤ *d* ≤ 0.79), or large (*d* ≥ 0.80). The α-value was *a priori* set at *p* < 0.05.

## Results

### Static balance performance

For all but one (i.e., EC-FO) stance condition, the ANOVA showed significant medium- to large-sized main effects of test (Table 3). A group × test interaction was only detected for the EO-FO stance condition (*p* = 0.009, *η*_p_^2^ = 0.15). Post-hoc tests revealed significant large-sized improvement for the BT-low-complex group (*t*=-4.685, *p* < 0.001, *d* = 1.09) but not for the BT-high-complex group (*t*=-1.151, *p* = 0.131, *d* = 0.20). The main effect of group did not reach significance.

**Table 3:** Effects of a 6-weeks balance training program with different levels of complexity

Test/outcome	BT-low-complex (*n* = 22)		Δ%	BT-high-complex (*n* = 22)		Δ%	*p*-value (η_*p*_^2^)		
	Pretest	Posttest		Pretest	Posttest		Test	Group × Test	Group
OLS									
OLS time; EO-FI [s]	48.5 ± 16.5	57.1 ± 8.9	15.1	53.4 ± 12.8	57.1 ± 8.0	6.5	**0.008 (0.16)**	0.277 (0.03)	0.392 (0.02)
OLS time; EC-FI [s]	15.5 ± 15.9	30.5 ± 18.6	49.2	19.9 ± 19.2	29.4 ± 19.2	32.3	**< 0.001 (0.42)**	0.220 (0.04)	0.740 (0.01)
OLS time; EO-FO [s]	31.8 ± 19.1	51.1 ± 15.5*	37.8	40.5 ± 20.7	44.6 ± 19.9	9.2	**< 0.001 (0.30)**	**0.009 (0.15)**	0.825 (0.01)
OLS time; EC-FO [s]	5.2 ± 2.2	6.4 ± 3.1	18.8	6.5 ± 4.8	6.2 ± 1.9	-4.8	0.405 (0.02)	0.160 (0.05)	0.467 (0.01)
3-m beam walk									
6.0-cm beam width [steps]	13.9 ± 2.8	15.9 ± 0.4	12.6	14.0 ± 3.3	15.3 ± 1.8	8.5	**< 0.001 (0.24)**	0.446 (0.01)	0.657 (0.01)
4.5-cm beam width [steps]	12.4 ± 2.8	14.1 ± 2.6	12.1	12.8 ± 3.1	14.6 ± 2.4	12.3	**0.002 (0.21)**	0.930 (0.01)	0.485 (0.01)
3.0-cm beam width [steps]	10.5 ± 3.9	11.9 ± 3.0	11.8	10.6 ± 3.2	13.6 ± 2.4	22.1	**< 0.001 (0.28)**	0.149 (0.05)	0.264 (0.03)
Total [steps]	36.8 ± 7.5	41.8 ± 5.0	12.0	37.4 ± 6.9	43.4 ± 4.2	13.8	**< 0.001 (0.44)**	0.605 (0.01)	0.488 (0.01)
YBT-LQ									
AT [% LL]	75.2 ± 7.6	78.1 ± 6.5	3.7	74.5 ± 6.2	76.6 ± 6.6	2.7	**0.003 (0.19)**	0.632 (0.01)	0.541 (0.01)
PM [% LL]	112.3 ± 10.2	116.4 ± 8.2	3.5	108.4 ± 9.0	115.6 ± 6.6	6.2	**< 0.001 (0.32)**	0.239 (0.03)	0.311 (0.02)
PL [% LL]	109.5 ± 9.0	113.7 ± 6.4	3.7	104.8 ± 8.9	110.6 ± 7.9	5.2	**< 0.001 (0.32)**	0.489 (0.01)	0.080 (0.07)
CS [% LL]	99.0 ± 8.0	102.7 ± 5.7	3.6	95.9 ± 7.3	100.9 ± 6.1	5.0	**< 0.001 (0.42)**	0.431 (0.02)	0.204 (0.04)

Data are presented as group mean values ± standard deviations. Values are *p*-values and effect sizes (*η*_p_^2^) in brackets with 0.02 ≤ *η*_p_^2^ ≤ 0.12 indicating small, 0.13 ≤ *η*_p_^2^ ≤ 0.25 indicating medium, and *η*_p_^2^ ≥ 0.26 indicating large effects. Bold values indicate statistically significant differences. *Indicates a significant differences between pretest and posttest

*AT* Anterior; *BT* Balance training; *CS* Composite score; *EC* Eyes closed; *EO* Eyes opened; *FI* Firm ground; *FO* Foam ground; *LL* Leg length; *OLS* One-Legged Stance test; *PL* posterolateral; *PM* Posteromedial; *YBT-LQ* Y- Balance-Test-Lower-Quarter

### Dynamic balance performance

Irrespective of beam width, the ANOVA showed significant medium- to large-sized main effects of test (Table 3). This indicates that improvements in step number were independent of the applied BT complexity. Neither the main effect of group nor the group × test interaction reached significance. Further, the ANOVA yielded significant medium- to large-sized main effects of test for all YBT-LQ reach parameters (Table 3). This implies that enhancements in reach distance were also independent of the used level of BT complexity. The main effect of group and the group × test interaction did not reach significance.

## Discussion

Consistent with our first hypothesis stating that both training regimen would be result in balance improvements, both groups significantly increased their static (OLS time) and dynamic (3-m Beam Walking Backward step number, YBT-LQ reach distance) balance performance. These findings are in line with those from previous studies [1, 2, 7, 29] that investigated the effect of BT in healthy male adolescents and indicate that BT is an effective method to enhance static and dynamic balance in youth. Adaptations at the spinal and supraspinal level are thought to be the underlying mechanisms [30].

Further, we hypothesised that a high versus low complex BT would lead to greater balance improvements. Contrary to that expectation, no significant group by test interactions were detected neither for static (except for the BT-low-complex group during the OLS test, EO-FO) nor for dynamic balance performance. This indicates that a low (i.e., single-motor-tasking) versus high (i.e., dual-motor-tasking) complex BT did not result in group-specific balance improvements. This result is consistent with the findings from a previous study [31] that conducted eight weeks of single- or dual-task BT in adolescents and reported no significant group by test interactions in measures of static (postural sway in the OLS test) and dynamic (gait parameters in the 10-m-walk test) balance. However, for the most difficult stance condition (OLS test; EC–FO) we did not detect significant enhancements indicating a ‘floor’ effect, i.e., the combination of supressed vision and modified proprioception was too challenging, which limited the potential for improvements.

What is the likely reason that the effectiveness of BT was not affected by differences in BT complexity? There is evidence [32, 33] that high versus low training complexity increases the capacity to perform multiple tasks simultaneously by reducing cognitive overload, i.e., freeing up central processing resources [9, 10]. Therefore, it seems likely that the applied configuration of high-complex BT was not sufficient to achieve concurrent task processing with reduced resources. Thus, subsequent studies should investigate whether a greater volume and/or different configuration of task complexity (e.g., triple-motor-tasking) will cause superior effects compared to the present version. Furthermore, it is known that dual-tasking is associated with interference (i.e., performance decrements in one or both of the executed tasks due to the concurrent use of processing resources) [34], which may have caused the lower improvements (see percent change in Table 3) in the BT-high complex group in some cases. In addition, balance assessments were only conducted under single-task conditions. For the group with the low complex BT, but not for the group with the high complex BT, this was identical to the training condition, which represents an advantage for the former in terms of task/training specificity [9]. Future studies should therefore perform assessment under both single- and dual-task conditions to confirm the task specific nature of BT induced adaptations.

## Conclusion

This study compared the effects of low (i.e., single-motor-tasking) versus high (i.e., dual-motor-tasking) BT complexity on measures of balance in youth. We observed significant improvements in static (i.e., OLS time) and dynamic (i.e., 3-m Beam Walking Backward step number and YBT-LQ reach distance) balance. However, the enhancements were not differentially affected by the applied BT complexity. These results imply that BT is an effective training regimen in healthy male adolescents but the applied high versus low complex BT exercises do not seem to provide additional effects.

### Limitations

- Only male adolescents were investigated, which limits the generalisation of findings to female adolescents.
- Field- but no laboratory-based testing (e.g., postural sway via force-plate) was used, which limits the internal validity.
- Effects of a mid-term BT regimen (6 weeks) were investigated, that cannot be transferred to long-term BT programs (i.e., lasting several months).
- Training-related changes were determined on a behavioural but not on a neuromuscular level (i.e., brain/muscle activity).

## Data Availability

The data generated and analysed during the present study are not publicly available due to ethical restrictions but are available from the corresponding author upon reasonable request.

## Abbreviations

ANOVA: Analysis of variance
AT: Anterior
BT: Balance training
CS: Composite score
EC: Eyes closed
EO: Eyes opened
FI: Firm ground
FO: Foam ground
FRT: Functional-Reach test
LL: Leg length
OLS: One-legged stance test
PHV: Peak height velocity
PL: Posterolateral
PM: Posteromedial
YBT-LQ: Y-Balance-Test-Lower-Quarter
